# Optimizing Acetabular Positioning: A Comprehensive Review of Contemporary Strategies in Total Hip Arthroplasty

**DOI:** 10.7759/cureus.59114

**Published:** 2024-04-26

**Authors:** Tarun Teja, Sandeep Shrivastava, Abhishek Choudhary, Vinit Rathod, Prashanth Balusani

**Affiliations:** 1 Orthopaedics, Jawaharlal Nehru Medical College, Datta Meghe Institute of Higher Education and Research, Wardha, IND

**Keywords:** orthopedic surgery, clinical outcomes, surgical techniques, contemporary strategies, acetabular positioning, total hip arthroplasty

## Abstract

Total hip arthroplasty (THA) is a widely practiced surgical intervention to alleviate pain and reinstate functionality in individuals afflicted with hip joint pathology. The positioning of the acetabulum assumes paramount significance in determining the efficacy of THA, exerting profound influences on biomechanical dynamics, stability, and the durability of outcomes over time. This comprehensive review meticulously evaluates contemporary methodologies for optimizing acetabular positioning in THA, encompassing advanced technologies such as computer-assisted navigation systems, patient-specific instrumentation, robotic-assisted surgical approaches, image-based planning techniques, and intraoperative fluoroscopy. Crucially, key discoveries underscore the pivotal role of precise acetabular alignment in mitigating complications such as dislocation, component wear, and impingement. Moreover, the implications for clinical practice accentuate the imperative of continuous education and training to ensure effective deployment of sophisticated methodologies. Recommendations for furthering research and enhancing practice development underscore the necessity of scrutinizing long-term prognoses, assessing cost-effectiveness, and embracing technological innovations perpetually refining THA outcomes. Collaborative endeavors among researchers, practitioners, and industry stakeholders emerge as indispensable drivers of advancement in this domain, fostering an environment conducive to elevating the standard of care for individuals undergoing THA.

## Introduction and background

Acetabular positioning holds paramount significance in total hip arthroplasty (THA), representing a cornerstone in achieving optimal outcomes for patients undergoing this procedure. The acetabulum serves as the socket portion of the hip joint, accommodating the femoral head and facilitating smooth, stable movement. During THA, the natural acetabulum is replaced with a prosthetic socket, necessitating precise placement to replicate normal hip biomechanics [1]. The positioning of the acetabular component directly influences the biomechanical function and stability of the artificial hip joint. Correct alignment and orientation are crucial for ensuring appropriate load distribution across the prosthetic surfaces, minimizing wear, and reducing the risk of implant loosening or failure. Proper placement also plays a critical role in restoring limb length, hip offset, and soft tissue tension, all of which contribute to optimal hip function and range of motion postoperatively [2].

Conversely, suboptimal acetabular positioning can harm the longevity and functionality of the prosthetic joint. Malpositioned components may result in impingement between the femoral neck and the acetabular rim, leading to pain, limited range of motion, and accelerated implant wear. Moreover, the acetabular component's inadequate coverage of the femoral head increases the risk of dislocation, a common complication following THA that significantly impacts patient satisfaction and quality of life [3].

Given the critical role of acetabular positioning in THA outcomes, meticulous attention to detail and precision are paramount during surgical planning and execution. Surgeons must consider various factors, including patient anatomy, biomechanics, and surgical approach, to achieve optimal component placement tailored to each patient. Advances in surgical techniques, navigation systems, and intraoperative imaging modalities have facilitated greater accuracy and reproducibility in acetabular positioning, enhancing THA procedures' overall success and longevity [2]. This review aims to comprehensively evaluate contemporary strategies for optimizing acetabular positioning in THA. By synthesizing current literature and examining advancements in surgical techniques, navigation systems, imaging modalities, and patient-specific instrumentation, this review aims to provide orthopedic surgeons with a thorough understanding of available options and their respective benefits and limitations. Additionally, the review seeks to analyze clinical outcomes and evidence supporting these contemporary approaches, offering insights into their effectiveness in improving patient outcomes and reducing complications. Through this comprehensive examination, the review intends to guide clinical decision-making and stimulate further research and innovation in THA.

## Review

Anatomy and biomechanics of the hip joint

Anatomy of the Acetabulum

The acetabulum, a sizeable hemispherical cavity located on the lateral aspect of the hip bone, forms a crucial component of the hip joint by articulating with the femoral head [4]. It results from the fusion of three pelvic bones: the ilium, ischium, and pubis. The ilium contributes to the upper boundary of the acetabulum, the ischium forms its posterior wall, and the pubis constitutes the anterior wall [5]. Structurally, the acetabulum comprises anterior and posterior columns, aiding in classifying acetabular fractures [5]. Its cup-shaped structure features a horseshoe-shaped lunate surface covered with hyaline cartilage, which interacts with the femoral head within the joint [6]. Additionally, the acetabular fossa, a central area devoid of an articular surface, is cushioned by a layer of fat and lined with a synovial membrane [6]. At its inferior aspect lies the acetabular notch, lacking defined margins and filled by the transverse acetabular ligament, a critical ligamentous structure [6]. The acetabular labrum provides stability and depth to the acetabular cavity, a fibrocartilaginous rim that encircles its margins [6].

As an integral hip joint component, the acetabulum participates in weight-bearing and locomotion, connecting the pelvis to the lower limb [5]. This ball-and-socket joint facilitates movement by allowing the spherical femoral head to rotate within its cup-shaped socket, enabling running, climbing, and jumping [5]. The articulation between the acetabulum and the femoral head, predominantly cartilaginous, ensures smooth movement within the joint [5]. Anteroinferiorly, the acetabular notch provides additional depth to the acetabulum [5]. Surrounding the acetabulum is the acetabular labrum, which enhances joint stability and depth [5].

Biomechanical Considerations in Acetabular Positioning

Biomechanical considerations play a pivotal role in THA success, particularly concerning the accurate positioning of the acetabular component. Achieving precise acetabular placement is crucial for restoring normal hip biomechanics, ensuring a functional range of motion, and mitigating potential complications like dislocation, muscle weakness, gait abnormalities, limb length disparities, impingement, noise generation, and implant wear and loosening [1,7]. Variables affecting acetabular cup position encompass depth, height, and angular positioning, including anteversion and inclination. Changes in the depth of the centre of rotation (COR) present implications for medialized versus anatomical positioning. While a medialized position may benefit joint reaction force, anatomical positioning offers advantages concerning range of motion, impingement, cortical rim press fit, and preservation of medial bone stock [1]. The height of the centre of rotation influences muscle activity, limb lengths, and available bone stock for cup support. The ideal angular position remains a subject of debate, with differing descriptions of angular position, including operative, radiologic, or anatomic definitions of anteversion and inclination. Pelvic tilt significantly influences the functional positions of the acetabulum, yet commonly employed positioning techniques may not accurately reflect the true orientation of the pelvis on the operating table. This discrepancy may result in significant pelvis adduction, flexion, and external rotation during surgery [1]. Recent technological advancements, such as 3D printing of device components and instrumentation, have improved THA outcomes. However, the success of THA is increasingly recognized as contingent upon achieving biomechanics tailored to each patient. Personalized orthopaedics, which involves tailoring healthcare based on genetics, lifestyle, and environment, is gaining momentum globally [7]. This personalized approach holds promise for further optimizing THA outcomes and enhancing patient satisfaction and functional outcomes.

Factors Influencing Optimal Acetabular Positioning

**Figure 1:**
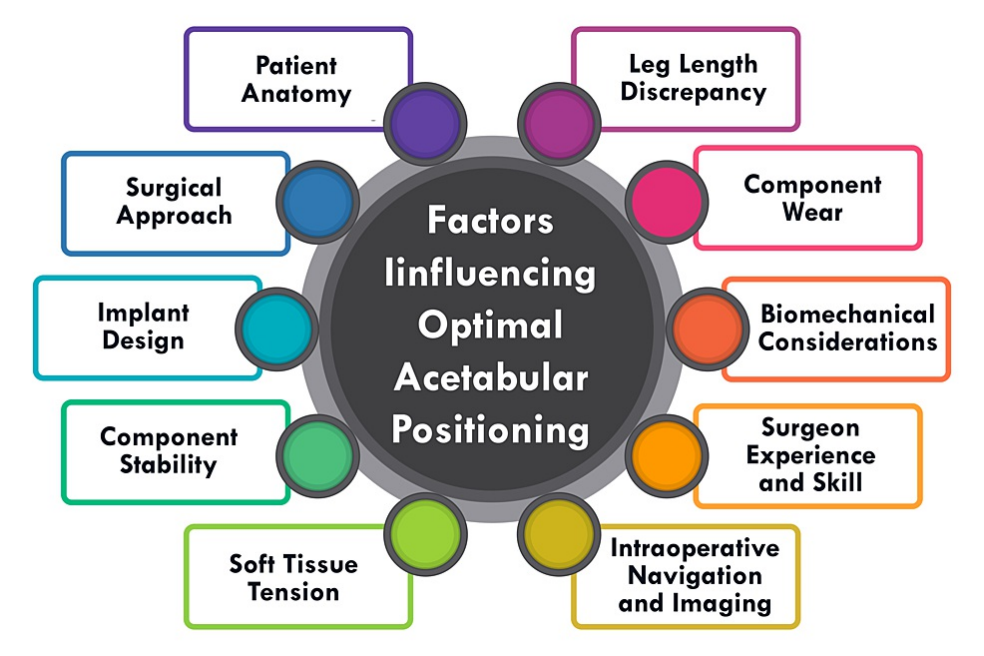
Shows factors influencing optimal acetabular positioning The image is created by the corresponding author.

Various factors influence optimal acetabular positioning in total hip arthroplasty, encompassing depth, height, angular position (anteversion and inclination), and pelvic tilt. Achieving precise placement of the acetabular cup is paramount due to its profound impact on numerous aspects of hip arthroplasty outcomes, including dislocation risk, abductor muscle strength, gait dynamics, limb length consistency, impingement potential, noise generation, range of motion, implant wear, loosening, and cup durability [1]. The depth of the acetabular component concerning the center of rotation affects joint reaction force and range of motion. At the same time, height influences muscle activation patterns, limb lengths, and the structural support provided to the cup by bone stock [1]. Angular positioning, encompassing anteversion and inclination, is pivotal in averting complications such as dislocation, accelerated wear, osteolysis, impingement, and limb length disparities [1,2]. Additionally, pelvic tilt emerges as a critical factor shaping functional acetabular positions, with variations in pelvic orientation exerting a significant influence on cup positioning during surgical intervention [1,2]. Achieving optimal acetabular positioning necessitates a comprehensive grasp of these multifaceted factors to enhance the efficacy and durability of total hip arthroplasty procedures. By meticulously considering and addressing each aspect, orthopaedic surgeons can strive to optimize patient outcomes and minimize the risk of complications associated with suboptimal acetabular placement. Figure 1 shows factors influencing optimal acetabular positioning.

Traditional approaches to acetabular positioning

Historical Perspective

The historical perspective on acetabular positioning in THA has evolved significantly. Initially, conventional THA procedures centred on medially positioning the prosthetic's COR relative to the native COR. This was typically achieved by medially placing the acetabular component and compensating for the increase through adjustments in femoral offset [8]. However, this approach presented challenges associated with soft tissue constraints, impingement issues, and instability due to the restriction of the joint's inherent range of motion [8]. A pivotal "kinematic revolution" has since transformed THA practice, shifting towards a more individualized consideration of patient biomechanics when determining implant placement strategies [8]. Surgeons have moved away from a standardized approach to acetabular cup placement, now prioritizing the restoration of a hip's native range of motion while respecting its inherent anteversion and ensuring precise, patient-specific cup positioning [8]. This paradigm shift underscores the increasing recognition of the importance of accurately reconstructing biomechanics to optimize joint function and implant longevity [2]. This evolution in THA reflects growing patient demands an enhanced range of motion, functional outcomes, and pain relief. Consequently, THA is increasingly offered to younger, more active individuals, underscoring the critical need to achieve longevity for the implant [1]. The transition towards patient-specific acetabular cup placement underlines the imperative of tailoring surgical interventions to individual biomechanical characteristics, aiming to optimize functional outcomes and implant durability in the face of evolving patient demographics and expectations.

Conventional Techniques and Their Limitations

Conventional techniques for acetabular positioning in THA have demonstrated notable limitations, notably involving the medialization or displacement of the COR [9]. These conventional methods may result in undesirable outcomes such as anterior overhang and potential impingement of the iliopsoas muscle or posterior overhang if excessive anteversion is pursued [9]. Suggestions to mitigate these issues include extended offset polyethene acetabular liners to preserve the hip COR. However, this approach may introduce heightened torsional forces at the liner-shell and bone-implant interface, thereby increasing the risk of loosening [9]. Recognizing the shortcomings of conventional techniques, there has been a notable shift towards more personalized approaches to acetabular positioning. Increasing recognition is given to the advantages of achieving an anatomically optimal position to restore hip biomechanics and functional range of motion [1,7]. The advent of robotic-assisted technology has further improved the precision of acetabular cup placement, often resulting in more cups implanted within the Lewinnek and Callanan safe zones [10]. Patient positioning is also a critical consideration in acetabular positioning during THA. While supine positioning simplifies the assessment of pelvic orientation and limb length, lateral decubitus positioning is more commonly employed, particularly with most surgeons' prevalent use of the direct anterior (minimally invasive) approach [8]. These advancements and considerations underscore the ongoing evolution towards personalized and precise techniques in acetabular positioning aimed at optimizing outcomes and minimizing complications in total hip arthroplasty.

Complications Associated With Suboptimal Positioning

**Figure 2:**
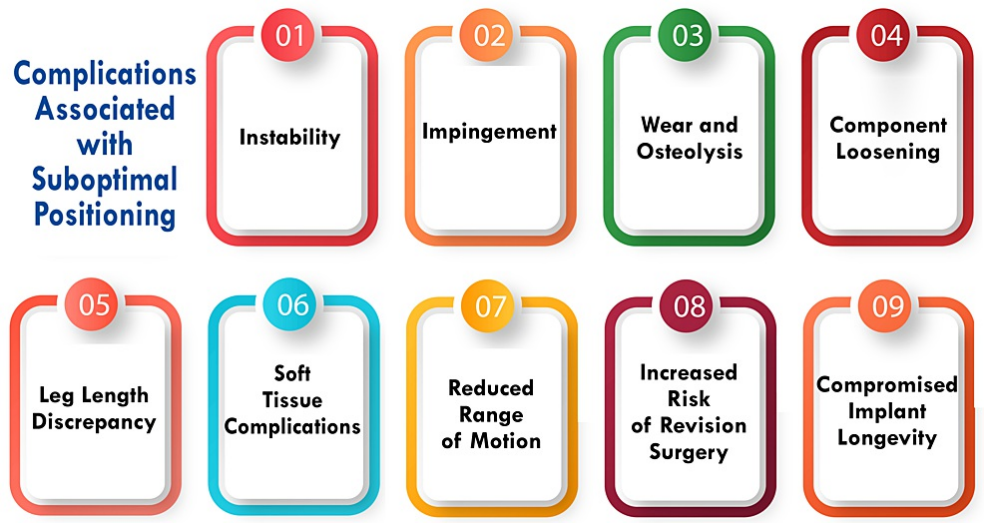
Shows complications associated with suboptimal positioning The image is created by the corresponding author.

Complications arising from suboptimal positioning of the acetabular component in total hip arthroplasty encompass a spectrum of issues, including a limited range of motion, heightened dislocation rates, accelerated wear of polyethene, metal-on-metal, and ceramic-on-ceramic bearings, fatigue fractures of highly cross-linked polyethene, squeaking of ceramic-on-ceramic bearings, ceramic fractures, poorer patient-reported outcomes, iliopsoas tendinitis, leg length discrepancies, compromised biomechanics, increased occurrences of osteolysis and aseptic loosening, component migration, and elevated revision rates [8,11-13]. These complications restrict the range of motion, exacerbate dislocation risk, and contribute to wear and fatigue-related failures across various bearing materials, impacting patient satisfaction and implant longevity [8,11-13]. Moreover, obese patients face heightened susceptibility to malpositioning compared to other BMI categories. This is attributable to factors such as a relatively diminished surgical field for a given incision size due to increased adipose tissue, alongside a substantial depth of fat that can influence the angle of the acetabular component inserter when encountering a deep wound [11]. Additionally, in obese patients, achieving optimal pelvic positioning can pose challenges, further predisposing to suboptimal placement of the acetabular component [11]. These considerations underscore the importance of tailored approaches and meticulous attention to detail in addressing patient-specific factors to optimize acetabular positioning and mitigate associated complications in total hip arthroplasty. Figure 2 shows complications associated with suboptimal positioning.

Contemporary strategies for acetabular positioning

Computer-Assisted Navigation Systems

Computer-assisted navigation systems represent a technological innovation utilized across various surgical specialities, including orthopaedic surgery, oral and maxillofacial surgery, and knee arthroplasty [14-16]. These systems leverage imaging technology to give surgeons real-time feedback, enabling more precise and accurate placement of implants and surgical instruments [17]. Comprising three primary components, computer-assisted navigation systems typically include a computer platform, a tracking system, and a rigid body marker [16]. The computer platform orchestrates the integration of inputs from the surgical field, interprets the data mathematically, and presents the resulting information on a monitor [16]. Meanwhile, the tracking system employs optical cameras, electromagnetic coils, or ultrasonic probes to capture signals from trackers affixed to the patient's bones or surgical instruments [16]. Computer-assisted systems can be categorized into three main types: active robotic systems, semi-active robotic systems, and passive systems [16]. Active robotic systems execute surgical tasks autonomously or restrict the surgeon's movements within predefined parameters, whereas semi-active systems furnish intraoperative information while allowing the surgeon discretion in decision-making [17]. Passive systems, such as navigation systems, display intraoperative data on a monitor while affording surgeons complete autonomy in decision-making [17]. While computer-assisted navigation systems offer numerous advantages, including real-time feedback and reduced intraoperative errors, several drawbacks and challenges merit consideration. These include the potential for increased surgical time and costs, the necessity for specialized training, and the susceptibility to technical issues or errors [17].

Patient-Specific Instrumentation

Patient-specific instrumentation (PSI) represents a technique employed in THA to refine the precision of implant positioning. This method entails the utilization of 3D-printed patient-specific guides meticulously crafted to optimize the placement of the acetabular component [18,19]. Notably, a study examining 100 consecutive patients demonstrated that implementing patient-specific guides resulted in highly accurate acetabular component placement, with 94.1% of cases falling within the planned position range of ± 10° [19]. PSI has been proven to enhance the accuracy of acetabular component placement, particularly in restoring implant orientation and mitigating leg length discrepancies [20]. A study examining 34 THA cases utilizing point-of-care manufactured patient-specific instruments revealed that 64.7% of patients could ambulate on the day of surgery with no reported complications [20]. Moreover, PSI can potentially optimize the surgical precision of component positioning, contributing to improved patient outcomes. This approach is a valuable and secure resource for surgeons navigating complex bone and joint deformities and cases [20]. Nevertheless, reliance on commercial PSI products may introduce potential challenges, such as increased procedural costs and extended waiting times for surgery [20]. Consequently, there is a growing shift towards point-of-care production of PSI, enabling more precise and safer THA procedures [20]. This evolution underscores the ongoing quest to enhance surgical accuracy and patient outcomes while addressing logistical and economic considerations.

Robotic-Assisted Surgery

Robotic-assisted surgery, also known as robot-assisted surgery or robotic surgery, revolutionizes surgical procedures by employing robotic systems. These advanced systems offer surgeons enhanced precision, flexibility, and control compared to traditional techniques, augmenting their capabilities and facilitating procedures in anatomically challenging regions through minimally invasive incisions [21-23]. In robot-assisted surgery, surgeons manipulate surgical instruments with unprecedented accuracy and skill, resulting in numerous benefits, including reduced complications, minimized pain and blood loss, shorter hospital stays, expedited recovery, and less conspicuous scarring [21]. The surgeon orchestrates the robotic arms through direct telemanipulation or computer control, providing real-time, high-definition, magnified, and three-dimensional views of the surgical field [23]. Commonly associated with minimally invasive approaches, robotic surgery offers additional advantages such as decreased infection risk, accelerated healing, and abbreviated patient hospitalization durations. Surgeons utilizing robotic systems commend the enhanced visualization and mastery afforded during procedures, enabling the execution of intricate and challenging manoeuvres that might otherwise prove formidable [24,25]. Despite the myriad benefits, robotic surgery entails risks akin to those encountered in conventional open procedures, including a slight infection risk and other potential complications. Patients must discuss comprehensively with their healthcare providers regarding robotic surgery's advantages and potential risks to ascertain its suitability for their medical requirements [25].

Image-Based Planning Techniques

Several advanced techniques are transforming pre-operative planning in THA, each offering unique benefits and considerations. One such approach involves 3D pre-operative planning, leveraging CT-based imaging and specialized software to predict implant size accurately, restore hip biomechanics, and determine component orientation. This method aims to enhance surgical accuracy, streamline implant inventory management, and facilitate the integration of computer-assisted techniques such as robotic-assisted surgeries and navigation systems [26,27]. Digital templating represents another evolving facet of preoperative planning, offering enhanced accuracy and reproducibility. Various methods provide clinicians with invaluable insights, including acetate templating on digital X-rays, digital 2D templating on digital X-rays, and 3D digital templating on CT scans. While 3D templating is recognized for its precision, evidence regarding its direct impact on clinical outcomes remains limited [28,29].

Integrating artificial intelligence (AI) technology further enhances preoperative planning capabilities, exemplified by software like AI HIP. This technology enables rapid and automatic acetabular and femur morphology identification, facilitating optimal prosthesis sizing. Studies suggest that AI-based planning enhances reliability in predicting component size in THA, considering factors such as acetabular dysplasia influencing accuracy [29]. Understanding the spine-hip relationship is paramount for optimizing acetabular cup orientation and overall biomechanical alignment. Parameters such as sacral slope, pelvic tilt, and pelvic incidence significantly influence spino-pelvic relations, impacting the hip joint's biomechanical setting [29]. Moreover, the advantages of 3D templating are increasingly recognized, particularly in complex cases. CT-based 3D templating offers superior accuracy to traditional methods, facilitating precise preoperative planning and mitigating surgical complications related to implant size and position. While its direct impact on clinical outcomes may vary, 3D templating is a valuable reference for bone landmarks, contributing to improved surgical outcomes [27,28].

Intraoperative Fluoroscopy

Intraoperative fluoroscopy is a pivotal medical imaging modality in orthopaedic procedures, including THA, to guide implant placement and alignment [30,31]. This technique offers real-time, continuous X-ray images on a monitor, enabling surgeons to visualize the patient's anatomy throughout the procedure [30,31]. In THA, fluoroscopy plays a crucial role in ensuring the precise positioning of the acetabular component, which is imperative for restoring joint function, enhancing joint stability, and mitigating complications such as leg length discrepancy, gait abnormalities, impingement, wear, and loosening [30,31]. Studies have demonstrated that integrating intraoperative fluoroscopy enhances the accuracy of acetabular cup placement and diminishes the incidence of associated complications [30,31]. However, intraoperative fluoroscopy entails exposure to ionizing radiation for both the patient and the operating room personnel, raising concerns regarding potential long-term health effects [32,33]. The extent of radiation exposure is influenced by various factors, including the procedure duration, the frequency of fluoroscopic image acquisition, and the proximity of the X-ray source to the patient or personnel [32,33]. Several strategies can be implemented to mitigate radiation exposure risks, including adopting low-dose fluoroscopy, optimizing the positioning of the X-ray source and the patient, and utilizing protective shielding for personnel [32,33]. Additionally, navigation technology has markedly reduced radiation exposure across all body regions for the operating surgeon and other operating room personnel compared to conventional fluoroscopy-guided procedures [32,33].

Three-Dimensional Printing Technologies

Several considerations are paramount in optimizing acetabular placement during THA. Firstly, surgeons should prioritize patient-customized placement by considering each patient's acetabular orientation and aiming to preserve native anteversion to restore the hip's inherent range of mobility [34]. Secondly, intraoperative considerations underscore the multifaceted nature of achieving accurate acetabular component placement, with factors such as medialization, depth, height, angular positioning, and pelvic tilt collectively influencing the success of THA [34]. The paradigm of orthopaedic medicine is evolving towards personalized orthopaedics, which tailors treatment strategies based on an individual's unique physiology, lifestyle, and environmental factors [35]. This approach acknowledges the importance of addressing patient-specific considerations to optimize outcomes in THA. Additionally, advancements such as 3D printing of device components and instrumentation contribute to improved outcomes by facilitating personalized treatment approaches [35].

Moreover, adopting the Kinematic alignment technique seeks to restore an individual's unique biomechanics when determining implant positioning, reflecting a shift towards patient-centred care in THA [36]. Furthermore, emerging technologies like the gravity-assisted guidance system offer a novel approach to ensuring reliable acetabular cup orientation during THA procedures, enhancing surgical precision and outcomes [36]. Finally, patient positioning plays a crucial role in THA, with supine positioning simplifying pelvic assessment and limb length evaluation. However, most surgeons perform THA with the patient in the lateral decubitus position, with some utilizing a direct anterior (minimally invasive) approach to achieve optimal surgical access [36]. These considerations underscore the multi-faceted approach required to optimize acetabular positioning and enhance outcomes in THA procedures.

Customized Implants

Customized implants represent a viable solution in revision total hip arthroplasty, particularly for addressing extensive acetabular bone loss [37]. A retrospective analysis evaluated the accuracy of custom-made acetabular implants by comparing preoperative planning with postoperative positioning in three patients who underwent acetabular custom-made prosthesis placement [37]. Custom designs were meticulously planned using 3D CT analysis, considering surgical focal points, and the accuracy of intended implant positioning was assessed by comparing pre-and postoperative CT scans, analyzing parameters such as the center of anteversion, inclination, screw placement, and implant-bone contact surface [37]. The analysis revealed satisfactory accuracy in implant positioning across the three cases, except malpositioning observed in the third case due to implant center of rotation posterization exceeding 10 mm [37]. All cases exhibited implant positioning within a difference of less than 10° of anteversion and inclination compared to the planned parameters [37].

In hip replacements, custom implants offer the potential for superior outcomes by precisely conforming to the individual's anatomy and replicating the natural hip joint with utmost accuracy [38]. This tailored approach can yield improved results, particularly in younger, more active patients who place greater demands on the implant [38]. Custom implants are meticulously designed and manufactured to restore equal leg length, optimize muscle function, and ensure the stability of the artificial ball and socket joint [38]. Moreover, custom-made femoral implants in total hip arthroplasty for congenital hip disease enhance stem-femur fit, redistribute strain, and reconstruct hip biomechanics [39]. A comprehensive review examines various aspects, including preoperative planning, design, material selection, surgical techniques, and clinical outcomes of using custom-made femoral implants in congenital hip disease patients [39]. Custom total hip replacement, or bespoke or patient-specific hip replacement, represents an advanced technology in total hip arthroplasty, utilizing an individualized hip implant tailored to the patient's unique anatomy [40]. This innovative approach necessitates preoperative CT scanning of the hip joint, enabling the creation of an implant that matches the patient's natural hip joint size and orientation [40].

Clinical outcomes and evidence

Review of Clinical Studies Comparing Traditional and Contemporary Techniques

The analysis of clinical studies comparing traditional and contemporary techniques underscores a notable trend toward embracing newer technology-based and virtual recruitment methodologies. These innovative approaches are designed to enhance research inclusivity by targeting more diverse populations while potentially expediting the recruitment process, thus offering distinct advantages in inclusiveness and efficiency [41]. Furthermore, there is a growing exploration of integrating technology into clinical trials to modernize research methodologies, with a specific emphasis on contrasting traditional and digital approaches to underscore the benefits of technological integration in research endeavours [42]. Comparing conventional and contemporary management control practices (MCP) in public health policies sheds light on differences in managerial approaches between individuals with clinical versus business backgrounds. Clinical-oriented managers are inclined to adopt MCP in a manner that fosters empowerment and collaboration. In contrast, their business-oriented counterparts may prioritize different management information and control techniques, potentially influencing strategic healthcare management decisions [43]. Moreover, within construction techniques, a comparative study evaluates traditional and contemporary building envelope construction methods, particularly in hot and humid climates. This research focuses on assessing thermal comfort and energy efficiency while exploring the feasibility of incorporating vernacular architecture principles into modern construction practices. The overarching goal is to promote sustainability and comfort in building design by drawing inspiration from traditional architectural practices [44].

Long-Term Outcomes and Survivorship Data

Evaluating the success of cancer treatments and the quality of life for survivors necessitates consideration of long-term outcomes and survivorship data. A study examining long-term outcomes in survivors of childhood cancer in India revealed a consistent decrease in the cumulative incidence of severe late effects, including mortality, in recent years [45]. Despite this positive trend, the study highlighted persistent psychosocial issues among survivors, such as neurocognitive impairment and metabolic syndrome. To comprehensively assess the physical and psychological effects of cancer treatment on survivors, the Patient Reported Outcomes Following Initial Treatment and Long-term Evaluation of Survivorship (PROFILES) registry is a valuable tool [46]. This registry collates data on patient-reported outcomes, encompassing aspects like physical function, emotional well-being, and overall quality of life. As cancer patients increasingly live longer, the field of survivorship research is rapidly expanding to address their evolving needs [47]. This includes tackling long-term adverse outcomes of cancer treatment, such as persistent neuropathy following treatment with taxanes [48]. Moreover, a study investigating the impact of cancer survivorship on the employment status of older workers revealed a higher likelihood of unemployment and lower earnings among cancer survivors [49]. The findings underscore the importance of implementing targeted interventions to support the employment needs of cancer survivors, thereby enhancing their overall well-being and quality of life.

Patient-Reported Outcomes

Patient-reported outcomes (PROs) are critical indicators of THA success, encompassing patient satisfaction, pain relief, and functional enhancement. A recent large, multicenter study in the United States compared PROs based on surgical approach for THA, revealing no significant difference in improvement between anterior and posterior approaches at three and six months postoperatively. However, patients undergoing the transgluteal approach exhibited significantly worse PRO improvement than those undergoing posterior approaches at the six-month mark [50]. Furthermore, primary hip preoperative factors, including age, sex, and preoperative pain, were found to influence PROs following THA significantly. Patients with higher preoperative pain scores demonstrated poorer PROs post-THA, whereas those with higher preoperative function scores exhibited better outcomes [51]. A review of PROs following THA indicated high patient satisfaction and functional improvement post-surgery, with most patients reporting substantial pain relief and enhanced physical functioning. Nonetheless, a subset of patients experienced persistent pain and dissatisfaction [50]. Additionally, a longitudinal cohort study delved into the role of cognitive appraisal processes in THA outcomes within the first year post-surgery. Findings revealed that specific appraisal processes, such as focusing on healthcare or living situation problems, were linked to worse outcomes in the initial six weeks post-THA. Conversely, other appraisal processes, like preparing one's family for health changes, correlated with improved outcomes during the same period [52]. In terms of short-term outcomes, a study examining the short-term results of total hip replacement (THR) identified patients experiencing functional recovery alongside those encountering treatment failure. While a majority of patients exhibited significant improvement in pain and functional outcomes following THR, a minority reported ongoing pain and functional limitations [53].

Challenges and future directions

Technical Challenges and Learning Curve Associated With Novel Techniques

Adopting novel techniques across various fields often involves significant technical challenges and a steep learning curve. These hurdles may stem from the intricate nature of the techniques, the requirement for specialized equipment or training, and the potential for unforeseen complications during implementation [54-56]. For instance, recent negative pressure wound therapy (NPWT) advancements have introduced innovations in negative pressure delivery, pumps, interface dressings, adhesive dressings, and tubing technology. Despite these strides, challenges persist in ensuring the optimal functioning of NPWT systems. Common issues include the failure to maintain negative pressure due to losing an airtight seal or tissue ingrowth into the interface dressing, leading to painful dressing changes and bleeding. Challenges like infection control and patient discomfort may also contribute to noncompliance with NPWT protocols [55].

Similarly, practitioners face various obstacles in technical writing, including delays in receiving reviews and feedback, inconsistent or outdated inputs, and reliance on complex, outdated, or unsuitable tools. Moreover, a lag in subject matter expert (SME) input and a lack of understanding of end users can further impede the document creation. These challenges often affect the translation department, typically at the end of the document creation life cycle [57]. To surmount these obstacles, technical writers can implement several strategies. These include sharing documents in advance to expedite the review process, involving the documentation department in planning from the outset, establishing a centralized documentation repository to streamline access to information, providing adequate training and funding for the writing department, and leveraging appropriate tools tailored to the task at hand [57].

Cost-Effectiveness Considerations

Cost-effectiveness considerations are pivotal in evaluating the efficacy of THA procedures. Numerous studies have underscored the cost-effectiveness of THA compared to non-operative management, particularly among patients over 80 years [58]. Primary THR cost-effectiveness has been well-established within specific patient cohorts, with an estimated 50,000 procedures performed annually in the UK alone [59]. Decision analytic models have been devised to gauge the cost-effectiveness of hip replacement procedures, factoring in variables such as implant expenses, revision rates, mortality rates, implications for quality of life, and overall healthcare expenditures [59]. In a comparative study contrasting total hip arthroplasty with resurfacing arthroplasty, the latter offered short-term efficiency advantages over the former within a selected patient demographic. Patients undergoing resurfacing arthroplasty reported higher quality of life and accrued additional quality-adjusted life years (QALYs) at a slightly higher cost [60]. The cost per QALY for resurfacing arthroplasty was deemed cost-effective within the initial 12 months of treatment, with the economic argument being more compelling for men than women [60]. Moreover, a five-year follow-up investigation assessing changes in quality of life and costs among patients undergoing primary total hip replacement through the Exeter Primary Outcomes Study revealed that patients gained approximately 0.8 QALYs over the five years. Notably, younger and male patients and those with lower body mass index and poorer hip scores exhibited significant associations with improved outcomes [61]. The cost per QALY for total hip replacement fell below accepted thresholds, indicating its cost-effectiveness compared to no surgery, with most cases remaining below the threshold of £20,000 per QALY [61].

Future Directions in Research and Technology

The future of enhancing artificial hip joints and their arthroplasty technologies is directed toward developing novel biomaterials capable of fostering a favourable immune environment at the implant site. These advancements promote better integration into the body, reducing the need for revision arthroplasty [62]. Furthermore, personalized orthopaedics, which tailors healthcare interventions based on individuals' genetics, lifestyle, and environmental factors, is gaining global momentum [62]. Recent technological strides, such as using 3D printing for fabricating device components and instrumentation, are contributing to improved outcomes in THA. However, the success of THA is increasingly perceived as contingent upon achieving biomechanics tailored to the unique characteristics of each patient [63]. In the realm of revision THA, acetabular reconstruction poses a formidable challenge, with each reconstructive method presenting distinct advantages and limitations [64]. Leveraging advanced MRI techniques holds promise in mitigating the challenges associated with MRI of hip arthroplasty, furnishing valuable insights for diagnosing and managing complications [63].

Potential Impact on Surgical Training and Education

The precise positioning of the acetabular component in THA is a pivotal focus in surgical training and education within orthopaedics. Orthopaedic surgeons meticulously consider the impact of acetabular positioning on patient-reported functional outcomes, cup placement accuracy, and polyethylene wear rates [65]. Among the various surgical approaches, the modified Hardinge approach emerges as one of the most frequently employed methods for THA, underscoring the importance of understanding its accuracy in orienting the acetabular component for optimal outcomes [65]. Intraoperative factors, including patient positioning and the utilization of navigation systems, significantly influence the precision of acetabular cup placement. Renowned surgeons contribute invaluable perspectives on managing patients with protrusion and dysplasia, shedding light on the role of navigation systems in achieving customized placement tailored to individual patients [8]. The paradigm shift toward a kinematic revolution in THA emphasizes the departure from standardized approaches to placement, instead prioritizing the restoration of a hip's inherent range of motion, thereby underlining the significance of individual biomechanics in clinical decision-making [8]. Structured postgraduate training profoundly influences hip surgery's learning curve, encompassing facets such as hip arthroplasty and acetabular positioning [66]. Furthermore, the adoption of acetabular positioning devices demonstrates promise in enhancing the accuracy of cup placement during primary total hip arthroplasty, thereby reducing variability and elevating overall surgical outcomes [67].

## Conclusions

In conclusion, this review underscores the critical importance of precise acetabular positioning in THA and examines contemporary strategies to optimize this surgical technique. Achieving optimal placement is emphasized in restoring hip biomechanics, ensuring stability, and maximizing long-term patient functional outcomes. Conversely, suboptimal positioning can lead to complications such as dislocation, component wear, and impingement, highlighting the necessity for meticulous attention during surgical planning and execution. These findings have significant implications for clinical practice, emphasizing the need for orthopedic surgeons to incorporate evidence-based approaches and leverage technological advancements to enhance patient outcomes. Furthermore, ongoing research is warranted to evaluate the long-term clinical outcomes, cost-effectiveness, and feasibility of contemporary techniques and to drive further innovation in navigation systems, imaging modalities, and patient-specific instrumentation. Collaborative efforts among researchers, clinicians, and industry partners are crucial to advancing the field and improving the quality of care for patients undergoing THA.
